# Fusobacterium nucleatum Pyogenic Liver Abscess and the Role of Bacterial Virulence and Gut Microbiota Dysbiosis

**DOI:** 10.7759/cureus.34548

**Published:** 2023-02-02

**Authors:** Scarlet F Louis-Jean, Nirav Agrawal, Sushrit Bisht

**Affiliations:** 1 Internal Medicine, Anne Arundel Medical Center, Annapolis, USA

**Keywords:** diverticulitis, bacterial virulence, gut microbiota dysbiosis, pyogenic liver abscess, fusobacterium nucleatum

## Abstract

In the United States, pyogenic liver abscesses are often due to monomicrobial infection and are rarely documented to be a consequence of *Fusobacterium *infection, a common cause of Lemierre’s syndrome. Recent advances in gut microbial studies have identified *Fusobacterium *as a commensal gut flora that becomes pathogenic in the setting of dysbiosis resulting from colorectal diseases, such as diverticulitis. While the bacteria’s tropism for the liver remains to be elucidated, the virulence pattern of *Fusobacterium* and the portal venous drainage system have allowed us to understand the bacterium’s propensity for causing right hepatic abscesses. In this case report, we detail an immunocompetent man with a history of sigmoid diverticulitis who developed a right hepatic abscess due to *Fusobacterium nucleatum*, while delineating a review of the literature on the virulent properties of the bacterium and the impact of gut microbiota dysbiosis in its pathogenicity. A descriptive analysis was also performed to identify the characteristics of patients who are at risk in hopes of further improving the clinical diagnostic schema for this condition.

## Introduction

Pyogenic liver abscess is an uncommon disease that is often polymicrobial and rarely monomicrobial [1]. With regard to liver abscesses, amebic organisms are among the most common causes worldwide [1]. In the United States, pyogenic causes of liver abscesses are often common, with a reported incidence of 0.55-2.3 per 100,000 [1,2]. Clinically significant infections often occur at a rate of 0.99 per 100,000 people per year [2]. It is purported that due to its rich vascularity and systemic and portal circulatory blood flow, the right lobe of the liver is a common site for visceral abscesses [1,3]. 

*Fusobacterium,* a non-spore forming facultative anaerobic gram-negative bacillus that is often part of the oropharyngeal flora, causes a range of invasive infections; however, based on the literature, pyogenic abscesses by this organism are rare [1,2]. Risk factors associated with the development of liver abscesses include immunosuppression, diabetes mellitus, hepatic transplant, intra-abdominal malignancy, and recent biliary tract procedures [4]. Although the mechanism by which hepatic inoculation of *Fusobacterium* is not entirely understood, it has been purported that gastrointestinal variants of this organism cause pyogenic liver abscesses [3]. Herein, we report a case of *Fusobacterium​*​​​​​​* nucleatum* pyogenic liver abscess in a middle-aged immunocompetent man and explore how *F. nucleatum* causes gut microbiota dysbiosis and gastrointestinal infection. We also include a descriptive analysis to help delineate risk factors and laboratory findings associated with pyogenic liver abscesses.

## Case presentation

A 43-year-old non-diabetic, immunocompetent Hispanic man with a history of anemia, recurrent sigmoid diverticulitis complicated by small intramural abscess, class 1 obesity, type I hypersensitivity to piperacillin-tazobactam, and hyperlipidemia presented to the emergency department reporting a five-day history of throbbing 10/10 right upper quadrant and epigastric abdominal pain, generalized body aches, fever, non-bloody non-bilious vomiting, inappetence, and five to six episodes of non-bloody and watery diarrhea. At the time of the encounter, the patient reported regularly eating outside meals but denied any recent travel history, consumption of new foods, intravenous drug use, alcohol misuse, or sick contacts. 

He was found to be afebrile with a temperature of 99.9 °F, normopneic with a respiratory rate of 20 breaths per minute, normoxic with an oxygen saturation of 96%, hypertensive at 138/52 mmHg, and tachycardic at 122 beats per minute. Physical exam was notable for bilateral rhonchi, and dry oral mucous membranes without evidence of decay, plaque, oropharyngeal exudates, or erythema. Additionally, there was localized left lower quadrant abdominal tenderness without rebound, guarding, rigidity, organomegaly, or ascites. Aside from an unremarkable complete blood count, laboratory results on admission were notable for potassium 3.2, creatinine 1.7, aspartate transaminase (AST) 124, alanine transaminase (ALT) 160, total bilirubin 2.5, serum lactate 4.7, procalcitonin 71.22, and alkaline phosphatase (ALP) 222 (Table 1). Imaging on admission included a chest x-ray, which was normal. The patient was initially presumed to have a viral syndrome in the setting of his constitutional symptoms and elevated liver transaminases. His upper respiratory panel was negative (Table 1). However, his hepatitis panel was notable for hepatitis A immunoglobulin G (IgG) antibodies (Table 1).

**Table 1:** Results of the patient’s diagnostic labs. Ab: antibody; Ag: antigen; ALT: alanine transaminase; AST: aspartate transaminase; BUN: blood urea nitrogen; Hep: hepatitis; IgG: immunoglobulin G; IgM: immunoglobulin M; pCO_2_: partial pressure of carbon dioxide; PCR: polymerase chain reaction; pH: potential hydrogen; pO_2_: partial pressure of oxygen; RSV: respiratory syncytial virus; SARS-COV-2: severe acute respiratory syndrome coronavirus 2; WBC: white blood cell.

Laboratories	Results	Reference ranges
Repeat complete blood count		
WBC count	16.97	3.40–10.80 mmol/μL
Differential		
Absolute neutrophils	14.66	1.80–7.70 × 10^3^/μL
Relative banded neutrophils	23	-
Atypical lymphocyte percent	1	-
Metamyelocytes percent	10	-
Myelocytes percent	7	-
Hemoglobin	11.8	13.5–17.0 g/dL
Hematocrit	36.1	41.0–51.0%
Platelets	104	140–400 mmol/uL
Basic metabolic panel		
Sodium	135	135–146 mmol/L
Potassium	3.2	3.5–5.2 mmol/L
Chloride	105	95–112 mmol/L
Bicarbonate	21	18–32 mmol/L
BUN	18	5–25 mg/dL
Creatinine	1.7	0.71–1.2 mg/dL
Serum lactate	4.7	0.5–2.0 mmol/L
Pro-calcitonin	71.22	≤0.10 ng/mL
Liver function test		
AST	124	10–40 U/L
ALT	160	4–44 IU/L
Total bilirubin	2.5	0.3–1.3 mg/dL
Alkaline phosphatase	222	32–132 IU/L
Total protein	5.4	6.4–8.2 g/dL
Albumin	1.7	3.2–5.0 g/dL
Hepatitis panel		
Hep A Ab, IgG	Reactive	-
Hep A Ab, IgM	Non-reactive	-
Hep B core Ab, IgM/IgG	Non-reactive	-
Hep B surface Ab, IgM/IgG	Non-reactive	-
Hep C Ab, IgM/IgG	Non-reactive	-
Cultures and miscellaneous		
Anaerobic culture (abscess aspirate)	Heavy *F. nucleatum*	-
Aerobic culture (abscess aspirate)	No growth after five days	-
Blood culture	No growth after five days	-
Urinalysis	Negative	
Urine culture	No growth after 18–24 hours	-
*Entamoeba histolytica* IgG	Negative	-
Lactate	4.7	0.5–2.0 mmol/L
Arterial blood gas analysis		
pH	7.46	7.35–7.45
pCO_2_	24.3	35.0–45.0 mmHg
pO_2_	62.7	90.0–100.0 mmHg
Upper respiratory PCR panel		
Adenovirus	Not detected	-
Coronavirus 229E	Not detected	-
Coronavirus HKU1	Not detected	-
Coronavirus NL63	Not detected	-
Coronavirus OC43	Not detected	-
SARS-COV-2	Not detected	-
Human metapneumovirus	Not detected	-
Human rhinovirus/enterovirus	Not detected	-
Influenza A PCR	Not detected	-
Influenza B PCR	Not detected	-
Parainfluenza 1	Not detected	-
Parainfluenza 2	Not detected	-
Parainfluenza 3	Not detected	-
Parainfluenza 4	Not detected	-
RSV PCR	Not detected	-
Bordetella pertussis	Not detected	-
Bordetella parapertussis	Not detected	-
*Chlamydia pneumoniae* PCR	Not detected	-
*Mycoplasma pneumoniae* PCR	Not detected	-

While on the general medical floor, the patient was managed with intravenous (IV) fluids for hypotension and lactic acidosis of 4.7, antiemetics, and monitored off antibiotics based on the initial suspicion that his presenting illness was due to a viral syndrome. One day into his admission, he developed a fever of 101.5 °F, chills, neutrophilic predominant leukocytosis with a white blood cell count (WBC) of 16.97, bandemia, and acute hypoxemic respiratory failure and acute non-anion gap metabolic acidosis with respiratory alkalosis as evidenced by an arterial blood gas pH of 7.46, partial pressure of carbon dioxide of 24.3 mmHg, and partial pressure of oxygen of 62.7 mmHg (Table 1). The patient was also hypotensive with a blood pressure of 85/56, although he maintained an appropriate mean arterial pressure of 66. His blood pressure remained unresponsive to a total of five liters of IV fluid boluses, which were administered over the course of the day to also mitigate his lactic acidosis (Table 1). He was then admitted to the intensive care unit (ICU) for vasopressor support in the setting of septic shock.

**Figure 1:**
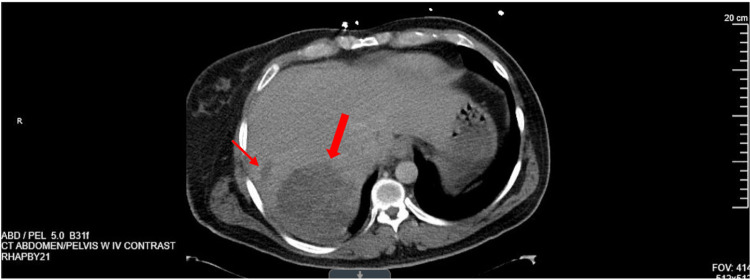
CT abdomen and pelvis without contrast demonstrating an indistinct hypodensity in the posterior segment of the right lobe, measuring 8.4 × 9.3 × 7.4 cm (large arrow), with an average density of 17 Hounsfield units, and a small ovoid hypodensity measuring 2.4 cm in the largest diameter (small arrow).

**Figure 2:**
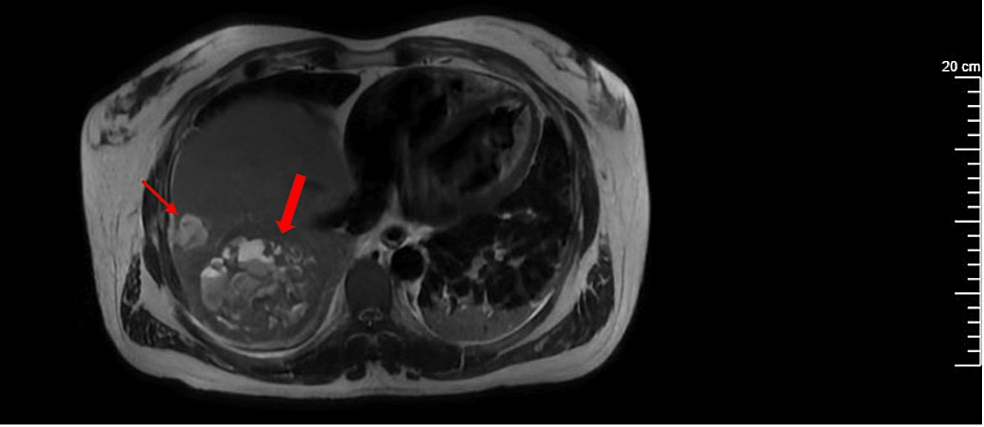
Transverse plane of MRCP without contrast demonstrating a heterogeneous lesion measuring approximately 9.0 × 8.2 × 8 cm (large arrow) at the right posterior segment of the right liver lobe near the dome, along with an anterolateral smaller complex lesion measuring 2.9 × 2.4 × 2.6 cm (small arrow). MRCP: magnetic resonance cholangiopancreatography.

Radiographic imaging was ordered upon transfer to the ICU. Ultrasound of the right upper quadrant demonstrated a 2.1 cm gallstone at the proximal neck of the gallbladder with a dilated common bile duct measuring 1.1 cm and trace pericholecystic fluid, which was thought to be choledocholithiasis with presumed ascending cholangitis. CT abdomen and pelvis (A/P) without contrast was later performed and demonstrated a large ill-defined hypodensity in the posterior aspect of the right liver, measuring 8.4 × 9.3 × 7.4 centimeters (cm) with an average density of 17 Hounsfield units, along with a small adjacent second hypodensity measuring 2.4 cm (Figure 1). Magnetic resonance cholangiopancreatography (MRCP) was recommended by the surgical team for further characterization, which confirmed the presence of the lesions presumed to be abscesses based on the patient's clinical picture (Figure 2). 

Sepsis work-up was initiated with urinalysis, urine culture, and blood culture along with immunoglobulin G for *Entamoeba histolytica*, which were all negative (Table 1). Transthoracic echocardiogram revealed a normal ejection fraction without evidence of vegetations. The patient was started on IV levofloxacin 500 mg daily due to a history of penicillin-induced anaphylaxis and metronidazole 500 mg three times daily (TID) per os (PO). At the time, it was suspected that the patient’s liver abscesses were resultant of cholangitis and a prior history of sigmoid diverticulitis. He then underwent CT-guided abscess drainage by interventional radiology, with 30 milliliters (mL) of foul-smelling fluid removed. Anaerobic culture of the abscess aspirate was notable for heavy gram-negative anaerobic rods, which speciated to *F. nucleatum* (Table 1). A repeat CT A/P, which was performed to evaluate for changes in the size of the lesions, showed no change but demonstrated existing sigmoid diverticulosis not initially addressed on prior scans and a new right-sided effusion, which appeared loculated.

Video-assisted thoracic surgery (VATS) decortication and chest tube placement were later performed by intrathoracic surgery for suspected complex effusion with early empyema; however, the drained fluid was serous in character and the fluid culture did not demonstrate evidence of WBCs or organisms. Several days later, the patient underwent laparoscopic cholecystectomy for pathology-confirmed chronic cholecystitis with cholelithiasis but without evidence of choledocholithiasis. On discharge, the patient was hemodynamically stable with improved abdominal pain and a normalized WBC count. Additionally, aside from an ALP of 237, the rest of his complete metabolic panel was within normal limits. He was given metronidazole 500 mg PO TID for three additional doses before departure and prescribed IV ertapenem 1 g every 24 hours for seven additional days to be administered at an infusion center, for a total treatment duration of 35 days, via a peripherally inserted central catheter (PICC) line. He was also given instructions to follow up with the infectious disease, surgery, and outpatient colonoscopy for further evaluation of his history of recurrent sigmoid diverticulitis and for colorectal cancer (CRC) screening. After 34 days, he underwent chest tube removal in the outpatient setting. It was documented that there was 15-20 mL of daily serous drainage appreciated while the chest tube was in place. 

## Discussion

Descriptive analysis

Background

In a review conducted in 2017, 48 cases of pyogenic liver abscesses were identified, of which 22 were secondary to *F. nucleatum* and *F. necrophorum* infections [2]. We conducted a descriptive analysis and identified that since the publication of the 2017 review article, there have been 15 additional published manuscripts, increasing the total of *F. nucleatum* (n=17) and *F. necrophorum* (n=20) pyogenic liver abscesses to 37 (Table 2).

**Table 2:** Table of demographic, laboratory, and causal data in patients with Fusobacterium liver abscess. AST: aspartate aminotransferase; ALT: alanine aminotransferase; ALP: alkaline phosphatase; CRP: C-reactive protein. *Recent dental procedure, poor dentition, peritonsillar abscess, and infectious mononucleosis. **Diverticular disease, colonic diverticulitis, sigmoid diverticulitis, and ulcerative colitis. ***Thrombosis of the portal vein branches, recent gynecological procedures, and hemorrhoidal band ligation.

Age (years)	Average	45
Sex	Male	30
	Female	7
Race	White	9
	African American	3
	Asian	5
	Unknown	20
Labs	AST (U/L)	47.25 (75.5–28.25)
	ALT (U/L)	43.75 (32.75–76.5)
	Total bilirubin (mg/dL)	2.78 (0.99–3.77)
	ALP (IU/L)	254 (158–412)
	CRP (mg/L)	180.275 (24.475–204.75)
Causes	Oropharynx*	15
	GI tract**	7
	Unknown	9
	Lemierre's syndrome	2
	Other***	4

Methods

A comprehensive literature search was conducted on PubMed, MEDLINE, and Google Scholar to include all studies related to pyogenic hepatic abscess and *F. nucleatum*. The medical subject headings (MeSH) terms used were hepatic abscess, adult, and *F. nucleatum*. A total of 50 studies were identified, of which 36 case reports and case series met the inclusion MeSH terms. The remaining studies were excluded as they were either written in different languages or did not provide access to the full article. Two independent reviewers assessed the literature to include all relevant studies, while a third reviewer helped resolve any articles with conflicts. 

Results

Based on the selected publications, the average patient age was 45 years and predominantly male (n=30; 81%) compared to female (n=7, 18.9%) (Table 2). Of the 36 cases, nine patients were white, three were African Americans, five were Asian, and 19 were of unknown race/ethnicity. Notable laboratory values, presented as interquartile ranges (Q1-Q3), included: AST 47.25 (28.25-75.5), ALT 43.75 (32.75-76.5), total bilirubin 2.78 (0.99-3.77), ALP 254 (158-412), and c-reactive protein (CRP) 180.28 (24.48-204.75) (Table 2). Nine of the 37 patients had non-reactive hepatitis panels, and two of them were hepatitis B positive (Table 3). A total of 22 patients had oropharyngeal or GI tract pathology, which included recent dental procedure, poor dentition, peritonsillar abscess, infectious mononucleosis, diverticular disease, colonic diverticulitis, sigmoid diverticulitis, and ulcerative colitis as the presumed primary sources of infection (Table 2). Additionally, four of the cases were sequelae of portal vein thrombosis, recent gynecological procedures, and hemorrhoidal band ligation, whereas two were due to Lemierre’s syndrome (Table 2). Nine of the cases did not have an identified source (Table 2).

**Table 3:** A delineation of individual characteristics, laboratories, identified organisms, and infectious sources in patients diagnosed with pyogenic liver disease. AA: African American; ALT: alanine transaminase; ALP: alkaline phosphatase; AST: aspartate aminotransferase; COPD: chronic obstructive pulmonary disease; CRP: C-reactive protein; ESRD: end-stage renal disease; F: female; HIV: human immunodeficiency virus; HTN: hypertension; M: male; No: number; Ref: reference.

No.	Ref	Year	Age	Sex	Race	Comorbidities	Labs						Organism	Source
							AST (U/L)	ALT (U/L)	Total bilirubin (mg/dL)	ALP (IU/L)	CRP (mg/dL)	Hepatitis panel		
1	[2]	2020	76	F	White	HTN and COPD	-	-	-	-	-	Negative	F. nucleatum	Unknown
2	[4]	2021	64	F	Unknown	Periodontal abscess	84	-	9	200	216	-	F. nucleatum	Oropharynx
3	[5]	2015	21	M	White	None	29	44	0.7	158	-	Negative	F. nucleatum	Oropharynx
4	[6]	2015	48	M	AA	HTN	74	99	2	204	21.52	-	F. nucleatum	Oropharynx
5	[7]	2015	23	M	Unknown	Unknown	-	-	-	-	-	-	F. nucleatum	Unknown
6	[8]	2014	58	M	Unknown	Unknown	-	-	-	-	-	-	F. nucleatum	GI tract
7	[9]	2012	48	M	AA	Unknown	74	59	1.3	-	-	Negative	F. nucleatum	Unknown
8	[10]	2011	49	M	Unknown	HTN	28	33	1.1	280	-	Negative	F. nucleatum	Oropharynx
9	[11]	2009	59	F	Asian	Unknown	-	-	-	-	-	-	F. nucleatum	Oropharynx
10	[12]	2008	58	M	White	ESRD	20	14	-	270	213	-	F. nucleatum	Oropharynx
11	[13]	2007	24	M	Unknown	None	33	38	-	128	-	Negative	F. nucleatum	Oropharynx
12	[14]	2005	62	M	Unknown	Ulcerative colitis	Elevated	-	-	-	-	-	F. nucleatum	GI tract
13	[15]	2001	78	F	White	Diverticular disease	-	21	0.88	356	-	-	F. nucleatum	GI tract
14	[16]	2001	68	M	Unknown	Unknown	Mild elevation	-	-	-	101	-	F. nucleatum	GI tract
15	[17]	1992	40	M	Asian	HIV	152	220	3.74	-	-	Hepatitis B positive	F. nucleatum	Unknown
16	[18]	1987	29	M	Unknown	None	-	-	-	-	-	-	F. nucleatum	Oropharynx
17	[19]	1984	70	M	Unknown	Unknown	Elevated	-	-	-	-	-	F. nucleatum	Unknown
18	[20]	2016	44	F	Unknown	Unknown	56	69	0.58	61	-	-	F. nucleatum	Unknown
19	[21]	2016	60	M	Unknown	HTN	Elevated	-	-	-	-	Negative	F. nucleatum	GI tract
20	[22]	2015	28	M	Asian	Unknown	-	-	-	499	28.67	-	F. necrophorum	Oropharynx
21	[23]	2013	30	M	AA	Unknown	152	114	2	115	-	-	F. necrophorum	Unknown
22	[24]	2012	44	M	Asian	Unknown	76	160	2.4	679	25	-	F. necrophorum	Lemierre's syndrome
23	[25]	2011	40	M	Asian	HTN	11	12	0.76	239	7.46	Negative	F. necrophorum	Unknown
24	[26]	2011	25	M	White	None	44	48	4.1	210	222	Negative	F. necrophorum	Oropharynx
25	[27]	2011	34	M	White	Unknown	-	-	-	-	-	-	F. necrophorum	Thrombosis of the portal vein branches
26	[28]	2009	21	M	Unknown	Unknown	18	30	-	-	-	-	*F. necrophorum *and *F. nucleatum*	Peritonsillar abscess
27	[29]	2009	25	M	Unknown	Hepatitis B and recent endodontic infection and periodontitis	-	32	-	138	24.3	Hepatitis B positive	F. necrophorum	Oropharynx
28	[30]	2007	64	M	Unknown	Unknown	Not elevated	Not elevated	Not elevated	Not elevated	171	-	F. necrophorum	Recent hemorrhoidal band ligation
29	[31]	2003	22	M	Unknown	Unknown	-	141	40	155	-	-	F. necrophorum	Lemierre's syndrome
30	[32]	2003	19	F	Unknown	Unknown	-	52	5.73	331	222	-	F. necrophorum	Gynecologic procedure and infectious mononucleosis
31	[33]	2002	71	M	White	Unknown	26	38	0.88	612	-	-	F. necrophorum	GI tract
32	[34]	1993	27	M	Unknown	Unknown	169	-	-	457	184	-	F. necrophorum	Oropharynx
33	[35]	1990	55	M	Unknown	Unknown	-	-	1.3	239	-	-	F. necrophorum	GI tract
34	[36]	1982	31	M	White	Unknown	Elevated	-	2.3	-	-	-	F. necrophorum	Unknown
35	[37]	1872	36	M	Unknown	Unknown	73	69	3.8	439	30.9	Negative	F. necrophorum	Oropharynx
36	[38]	2016	57	M	White	None	31	61	-	412	-	-	F. necrophorum	Oropharynx

Bacterial pathogenicity 

*Fusobacterium* is a non-sporulating obligate anaerobic filamentous gram-negative rod that belongs to the phylum* Fusobacteria* [39]. Within the Fusobacteriaceae family, there are nine genera, which include *Fusobacterium* and *Leptotrichia* [40]. Within the *Fusobacterium* genus, there are 14 species that have been known to be pathogenic to man and animals, among which *F. nucleatum* and *F. necrophorum* are the most isolated bacteria [40]. *F. nucleatum* is often known to be a component of oral plaque due to its ability to form biofilm, whereas *F. necrophorum* is usually found in the appendix of the colon [2,40]. Recent developments have discovered that *F. nucleatum* is a more common microbial inhabitant of the GI tract than originally believed [40]. In a study that conducted a culture-based survey of gut biopsies from a cohort of 59 patients, a total of 16 *Fusobacterium* species, most of which were *F. nucleatum*, were isolated [40]. Many of the isolates were discovered in patients with a history of inflammatory bowel disease (IBD), specifically Crohn’s disease [40]. 

The infectivity of the bacterium depends on its virulence properties, which in the case of *Fusobacterium* include *Fusobacterium* adhesin A (FadA), fatty-acid-binding protein 2 (Fap2), and mucin 2 (MUC2) proteins. FadA is an important 129 amino acid protein that allows *Fusobacterium* to inhabit the oral mucosa, which can explain the frequency of odontological diseases associated with the bacteria [41]. FadA serves to both provide attachment and invasion of the host cells via proteolysis [40]. It also participates in the evasion of the host defense system and has been shown to bind E-cadherin, a cell junction molecule on colorectal cells [40,42]. The organism’s attachment to these cell junctions allows for direct cellular and peri-cellular invasion, increased endothelial permeability, and systemic dissemination [43]. Colonization and invasion of the bacterium are also dependent on the production of the MUC2 protein, which is necessary for the synthesis of biofilm [40]. Mucin is implicated in the disruption of the mucosal barrier and exposure of underlying epithelial cells, which promotes the invasion of *F. nucleatum* and its co-aggregation with other microbial organisms [40]. Fap2 also plays an essential role in invasion as it is an adhesion protein that attaches to the host’s cell surface [44]. 

In the case of clinical infection, *Fusobacterium* was first identified as a cause of bacteremia in 1936 by Lemierre in the setting of an oropharyngeal infection causing internal jugular venous thrombophlebitis [45]. This condition was later eponymously named Lemierre syndrome [45]. Recent literature has identified a gastrointestinal variant of Lemierre syndrome involving the formation of septic thrombophlebitis of the portal vein due to infection with *F. nucleatum* [46]. These cases of pyogenic liver abscess, however, have been a consequence of ischemia, bacterial translocation via the portal vein, malignancy, foreign body, or post-traumatic or post-surgical inoculation [46]. Moreover, *F. nucleatum* is increasingly being recognized as an onco-bacterium involved in the tumor microenvironment of colorectal cancer, owing to the presence of the above-mentioned virulence factors [47]. Thus, the manifestation of systemic infection by this bacterium along with other anaerobic organisms warrants further work-up for malignancy.

Gut microbiota dysbiosis and case evaluation

The gut microbiome plays an essential role in modulating human health and disease. Facilitated by its virulence proteins FadA and Fap2, part of the pathogenic nature of the bacteria involves its ability to cause gut microbial dysbiosis through epithelial barrier disruption, a process that is propagated by the release of proinflammatory cytokines such as interleukin-8 and tumor necrosis factor [48,49]. This in turn leads to visceral hypersensitivity and colonic inflammation, both of which are processes identified in the development of irritable bowel syndrome (IBS), inflammatory bowel disease (IBD), and colorectal cancer (CRC) [48]. Several studies have also identified an increase in the abundance of *Fusobacterium* in the intestinal tracts of individuals with liver cirrhosis, primary sclerosing cholangitis, gastroesophageal reflux disease, HIV infection, and alcoholism [50]. Patients with IBD have exhibited a higher level of *F. nucleatum* in the colon and are therefore at significantly higher risk for CRC [51]. The possibility of colorectal cancer was not pursued in our patient due to a lack of radiographic evidence of malignancy and diagnostic anchoring. Nevertheless, the patient was instructed to follow up for CRC screening in the outpatient setting.

Diverticulitis has also been implicated in gut microbiota dysbiosis, a history of which our patient had, complicated by a history of a small intramural abscess. Diverticulitis is considered an infection of an outpouching of colonic mucosa and submucosa that is triggered by a disruption in microbial symbiosis [52]. The precursor to diverticulitis is the presence of diverticulosis, but the precipitant of this condition remains elusive, although theories have suggested that disruption of the gut microbiota is associated with environmental factors, obesity, and chronic constipation [53]. It has been postulated that dysbiosis of the microbiome within a diverticulum lowers the threshold for the development of diverticulitis, the risk of which is further increased by fecalith impaction [53]. Pyogenic liver abscess resultant of diverticulitis is a rare phenomenon but has occurred in the setting of portal vein pyemia, translocation from the mesenteric veins to the portal circulation, or bacteremia in the setting of micro-perforation [43]. Since our patient did not have any active signs of simple or complicated diverticulitis, this was unlikely the source, but this was presumed to be the etiology of his pyogenic liver infection as there were no other identified sources of infection at the time.

In the case of our patient, obesity and a westernized diet could have also played a role, especially as emerging data have shown that these two factors have a correlation with an increased ratio of pathogenic gut bacteria. This disruption in turn leads to inflammation and metabolic disturbances involved in many diseases. Our patient endorsed frequent consumption of restaurant foods and takeout; however, a more detailed dietary history was not pursued. Nonetheless, based on a limited social history, we determined that the patient was a never-smoker with six years of sobriety from alcohol use, both of which are behavioral factors that are associated with gut microbiota dysbiosis. There was also some suspicion of cholangitis as an etiology, but this was determined to be less likely as the patient’s clinical progression and post-cholecystectomy pathology were not consistent with this diagnosis. 

Due to the rarity of pyogenic liver abscess, its diagnosis is likely to be missed by medical providers. In the case of our patient, his presumed viral illness delayed the appropriate work-up, and this ultimately led to septic shock. Several case reports have attempted to identify markers or clinical findings to support the inclusion of *Fusobacterium* liver abscess in the armamentarium of differentials in patients presenting with right upper quadrant abdominal pain, diverticulosis, or poor oral hygiene; however, no definitive criteria have been established. We conducted a descriptive analysis to identify risk factors and lend to the growing body of literature on pyogenic liver abscesses. However, our analysis was limited by its small sample size and overall lack of power needed to identify strengths of association and statistical significance. Additionally, the heterogeneity of the laboratory findings, comorbidities, and demographics made it difficult to identify a more robust list of risk factors. Nonetheless, it appears that common risk factors for *Fusobacterium* pyogenic liver abscesses include men in their fourth decade of life with a history of odontological/periodontal and GI tract pathology. 

## Conclusions

Owing to the rarity of this condition, the diagnosis of monomicrobial pyogenic liver abscess is difficult for clinicians to make. Certain risk factors increase predisposition to the disease, which include being middle-aged, male, and having a history of oropharyngeal or gastrointestinal pathologies or recent intervention. Due to a paucity of specific diagnostic criteria and an increased likelihood for adverse outcomes, clinicians should conduct a thorough history and physical assessment and order appropriate diagnostic tests for early disease identification. Additionally, it is recommended that patients presenting with *Fusobacterium* liver abscess should undergo cancer screening as *Fusobacterium *is being increasingly identified in the tumor microenvironment of CRC.
